# Innovative linear purse-string suture technique for closure of post-endoscopic submucosal dissection rectal defect

**DOI:** 10.1055/a-2628-8951

**Published:** 2025-07-15

**Authors:** Zhongshang Sun, Tianheng Ma, Xin Liu, Shuran Hu, Rui Xie

**Affiliations:** 1 Department of Gastroenterology, The Affiliated Huaian No.1 Peopleʼs Hospital of Nanjing Medical University, Huaian, China; 2 Department of TCM Tui Na Therapy, Huaian TCM Hospital Affiliated to Nanjing University of Chinese Medicine, Huaian, China

A 78-year-old female patient presented with a rectal laterally spreading tumor. Following standard endoscopic submucosal dissection (ESD), we initially planned for conventional purse-string suturing 1 , the linear morphology of the defect prompted the adoption of a modified linear technique to overcome inherent limitations (inadequate closure and prolonged duration).

The procedure commenced with the deployment of a clip to anchor the nylon loop at the proximal edge of the defect. Subsequently, a second clip secured the dual-strand nylon loop posterior and rightward to the initial anchor point, followed by the sequential placement of a third clip retracting and fixing the loop posterior and leftward to the second clip to initiate defect linearization. A fourth clip was then positioned posterior and rightward to the third clip, further enhancing linear tension alignment. This alternating left–right fixation pattern was iteratively applied until complete linear coaptation of the defect margins was achieved. Finally, under continuous endoscopic visualization, the nylon loop was cinched to complete the suture, ensuring optimal mucosal apposition without residual gaps ( Fig. 1 , Fig. 2 , Video 1 ).

**Fig. 1:**
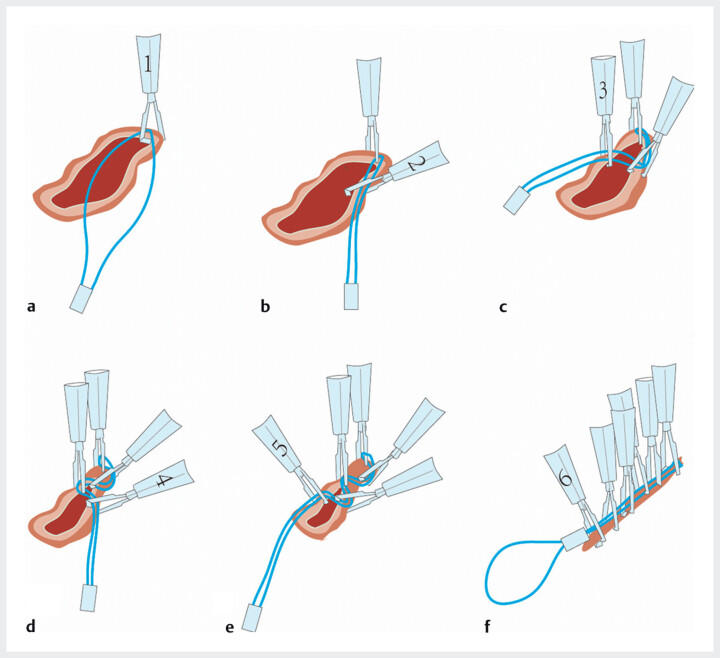
Linear purse-string suturing achieves endoscopic closure of rectal defects. **a** The nylon loop was initially secured at the proximal margin of the defect using a clip. **b** A subsequent clip was applied to affix the dual-strand nylon loop posteriorly and to the right of the initial anchor point. **c** A third clip was then sequentially positioned posteriorly and leftward relative to the second clip, retracting and securing the loop to initiate linear alignment of the defect. **d** The fourth clip was positioned posterior and rightward relative to the third clip to further align linear tension vectors. **e** Sequential alternating left–right clip placement enabled uniform tension distribution, resulting in complete linear coaptation of the defect. **f** Final tightening of the nylon loop was performed under endoscopic guidance, achieving optimal mucosal alignment without residual gaps.

**Fig. 2:**
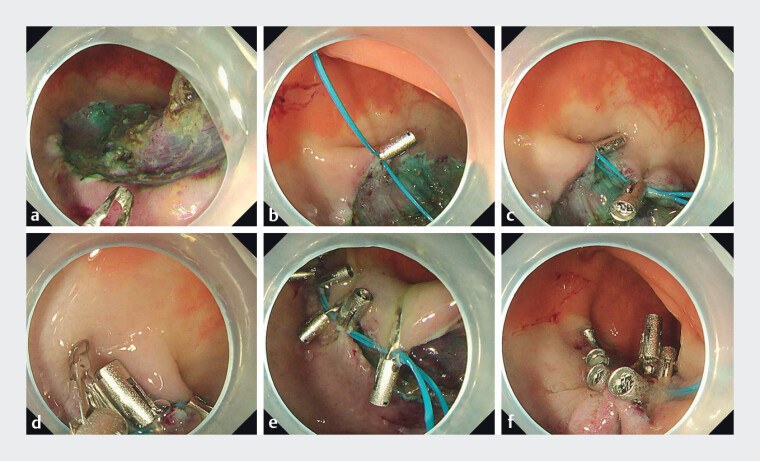
Endoscopic rectal defect closure via linear purse-string suture. **a** Purse-string suture was particularly challenging for elongated defects with high length-to-width ratios. **b** A clip anchored the nylon loop at the proximal defect edge. **c** A second clip fixed the double nylon loop posterior-rightward to the anchor point. **d** Subsequent placement of a third clip posterior-leftward to the second clip. **e** Alternating left–right fixation achieved complete defect coaptation. **f** The nylon loop was cinched, ensuring mucosal apposition without gaps.

Linear purse-string suture for post-endoscopic submucosal dissection rectal defect closure.Video 1

The technical innovation of this modified linear purse-string suture lies in its ability to address anatomical challenges specific to elongated post-ESD defects, where traditional circular closure methods risk “dog-ear” deformities and incomplete sealing due to excessive length-to-width ratios. By implementing an alternating clip fixation strategy, directional tension forces were strategically redistributed from circumferential to linear vectors, optimizing tissue apposition. Based on our center’s clinical experience, this approach demonstrated a 40% reduction in operative time compared to conventional techniques, with postprocedural 48-hour follow-up confirming 100% primary closure integrity, effectively mitigating leakage risks. The linear configuration specifically counters the geometric constraints of longitudinal defects, where uneven tension distribution historically compromised closure efficacy.

Endoscopy_UCTN_Code_TTT_1AQ_2AK

## Notes

Endoscopy E-Videos https://eref.thieme.de/e-videos
*E-Videos* is an open access online section of the journal *Endoscopy* , reporting on interesting cases and new techniques in gastroenterological endoscopy. All papers include a high-quality video and are published with a Creative Commons CC-BY license. Endoscopy E-Videos qualify for HINARI discounts and waivers and eligibility is automatically checked during the submission process. We grant 100% waivers to articles whose corresponding authors are based in Group A countries and 50% waivers to those who are based in Group B countries as classified by Research4Life (see: https://www.research4life.org/access/eligibility/ ). This section has its own submission website at https://mc.manuscriptcentral.com/e-videos .
